# A comprehensive longitudinal study of gut microbiota dynamic changes in laying hens at four growth stages prior to egg production

**DOI:** 10.5713/ab.23.0271

**Published:** 2023-10-21

**Authors:** Seojin Choi, Eun Bae Kim

**Affiliations:** 1 Department of Applied Animal Science, College of Animal Life Sciences, Kangwon National University, Chuncheon, 24341, Korea; 2 Institute of Animal Life Science, Kangwon National University, Chuncheon, 24341, Korea

**Keywords:** Gut Microbiota, Laying Hens, Longitudinal Study, 16S rRNA Gene

## Abstract

**Objective:**

The poultry industry is a primary source of animal protein worldwide. The gut microbiota of poultry birds, such as chickens and ducks, is critical in maintaining their health, growth, and productivity. This study aimed to identify longitudinal changes in the gut microbiota of laying hens from birth to the pre-laying stage.

**Methods:**

From a total of 80 Hy-Line Brown laying hens, birds were selected based on weight at equal intervals to collect feces (n = 20 per growth) and ileal contents (n = 10 per growth) for each growth stage (days 10, 21, 58, and 101). The V4 regions of the 16S rRNA gene were amplified after extracting DNA from feces and ileal contents. Amplicon sequencing was performed using Illumina, followed by analysis.

**Results:**

Microbial diversity increased with growth stages, regardless of sampling sites. Microbial community analysis indicated that Firmicutes, Proteobacteria, and Bacteroidetes were the dominant phyla in the feces and ileal. The abundance of *Lactobacillus* was highest on day 10, and that of *Escherichia-shigella* was higher on day 21 than those at the other stages at the genus level (for the feces and ileal contents; p<0.05). Furthermore, *Turicibacter* was the most abundant genus after changing feed (for the feces and ileal contents; p<0.05). The fecal *Ruminococcus torques* and ileal *Lysinibacillus* were negatively correlated with the body weights of chickens (p<0.05).

**Conclusion:**

The gut microbiota of laying hens changes during the four growth stages, and interactions between microbiota and feed may be present. Our findings provide valuable data for understanding the gut microbiota of laying hens at various growth stages and future applied studies.

## INTRODUCTION

The poultry industry is an essential part of the agricultural sector. It plays a crucial role in providing a considerable source of animal protein, such as meat and eggs, to meet the growing demands of the global population [1]. In the poultry industry, the microbiome of poultry birds is critical in determining their health, growth, and productivity [2].

The microbiota is the community of microorganisms living in a particular environment, such as bacteria, fungi, and viruses. The connection between the human microbiome and health has been studied for over a century [3]. Recently, attention has been paid to the significance of the association between livestock and microbiota. Previous research has investigated fluctuations in the gut microbiota throughout lactation and weaning in pigs [4] and the rumen microbiota in sheep and its impact on feed efficiency [5]. The gut microbiota is the community of microorganisms living in the digestive tract of animals and is indispensable for efficient nutrient digestion and absorption. Furthermore, it plays a vital role in defending the host against pathogenic microorganisms [6]. The gut microbiota changes over time in animals [7].

This study selected laying hens (Hy-Line Brown), the most balanced brown egg layer worldwide. The importance of the gut microbiota in laying hens, as in other livestock, is gaining attention. A healthy microbiota in laying hens can reduce the risk of food-borne diseases [8]. Furthermore, a thorough understanding of gut microbiota changes over time is required to select the appropriate feed additive, such as probiotics and prebiotics [9], for each stage of chicken growth to achieve healthy eggs. However, there have been limited studies on the basic physiology of livestock, and few studies have investigated the intestinal microbiota in laying hens at different growth stages. We suggest that the gut microbiota of laying hens undergoes dynamic changes over time and can serve as an important indicator of the overall health and development of the gut microbiome. Therefore, changes in the gut microbiota of laying hens were monitored from birth to the pre-laying stage.

## MATERIALS AND METHODS

### Animal trial

Laying hens (Hy-Line Brown, n = 80) were obtained from poultry farms (Korea Poultry Co., Anseong, Korea) and raised on a farm in Chuncheon, Korea, following Korean animal welfare guidelines. Birds were fed a commercial diet suitable for their growth stages. Starter and well-textured mash diets were provided *ad libitum* on days 0 to 43 and 44 to 101 (Table 1), with free access to water. A microcontroller (NodeMCU) continuously monitored the temperature and humidity. All experiments were approved by the Institutional Animal Care and Use Committee (IACUC) of Kangwon National University (KW-220425-5).

### Sample collection

Twenty birds were selected based on body weights at each sampling point, spaced at equal intervals. For feces collection, the birds were isolated in a clean plastic pen floored with sterilized aluminum foil and weighed. Ten birds were selected from the twenty whose feces had been collected, spaced at equal intervals, and euthanized by CO_2_ asphyxiation to collect ileal contents at 10, 21, 58, and 101 days (Supplementary Table S1). In short, 1 g of feces and ileal contents were placed into 1.7 mL tubes using a sterilized tip. In total, 80 fecal and 39 ileal content samples were stored at −70°C, until DNA extraction.

### DNA extraction and 16S rRNA sequencing

DNA was extracted from 250 mg feces and ileal contents using the NucleoSpin Soil kit (Macherey–Nagel, Düren, Germany). Briefly, each sample was homogenized using 0.6 to 0.8 mm ceramic beads in NucleoSpin bead tubes and a Taco Prep bead beater (GeneReseach Biotechnology Corp., Taichung, Taiwan). Subsequently, DNA was extracted following the manufacturer’s instructions. The extracted DNA was stored at −20°C until further analysis. The V4 region of the 16S rRNA gene was amplified using TaKaRa Ex-Taq polymerase (TaKaRa Bio, Shiga, Japan) and universal primers (forward: 5′-GGA CTACHVG GGTWTCTAAT-3′ and reverse: 5′-GTGCC AGCMGCCGC GGTA A-3′) with the following amplification conditions: 94°C for 3 min, followed by 30 cycles at 94°C for 45 s, 55°C for 1 min, 72°C for 1.5 min, and finally at 72°C for 10 min [10]. Amplicons were purified using a QIAquick polymerase chain reaction (PCR) purification kit and normalized to 50 ng per sample using a Spark 10 M Multimode microplate reader (Tecan Group AG, Männedorf, Switzerland). DNA library construction and sequencing were performed using the Illumina MiSeq platform (eGenome Inc., Seoul, Korea) to generate paired-end reads of 2×250 bp.

### Microbiome analysis

Quantitative Insights into Microbial Ecology 2 (QIIME 2) v.2021.4 (https://qiime2.org) and the SILVA 16S rRNA gene reference database were used to analyze microbiome communities [11]. Individual primers and adapters were trimmed from raw sequencing reads using the QIIME2 plugins cutadapt and demux and demultiplexed using in-house Perl scripts [12]. Demultiplexed reads underwent quality trimming, filtering, and chimeric sequence removal using the denoise-paired option in the Divisive Amplicon Denosiong Algorithm (DADA) 2 plugin [13]. The DADA2 denoise-pair options were as follows: 8 base pairs from the left were trimmed and truncated at 180 bp. The generated phylogenetic tree was used to analyze the microbial diversity of the samples. Multiple alpha and beta diversity indices were generated from the phylogenetic tree using core-metrics-phylogenetic, alpha-group-significance, and beta-group-significance options in QIIME 2.

Alpha diversity (Shannon, Faith’s phylogenetic distance, and Pielou’s evenness) was used to evaluate species richness, evenness, and phylogenetic distance of the microbiota. Beta diversity was assessed using nonmetric multidimensional scaling (NMDS) using the Bray–Curtis dissimilarity matrix between samples, which was conducted using the vegan package in R. NMDS is an ordination technique used to visualize data patterns in N-dimensional spaces. Adonis statistical tests utilizing 999 permutations were employed to evaluate how innate factors affect the microbial community during the growth stages. The amplicon sequence variants generated by DADA2 were assigned to taxonomic classifications using the SILVA 132 16S rRNA classifier and pre-trained using the QIIME 2 fit-classifier-naïve Bayes option.

### Metagenomics prediction

Metagenomics function was predicted using Phylogenetic Investigation of Communities by Reconstruction of Unobserved States (PICRUSt) that, which utilizes 16S rRNA marker gene sequences and references to previously published complete genome sequences [14]. The 16S rRNA gene copy numbers in the BIOM files were normalized and adjusted, and the metagenomes were predicted using precomputed Kyoto encyclopedia of genes and genomes (KEGG) orthologs. The predicted metagenomes were then aggregated based on a specific hierarchy level in the KEGG pathway using its metadata. Principle component analysis (PCA) was performed using STAMP v.2.1.3.

### Statistical analysis

Statistical analyses were performed using R v.4.1.3. One-way analysis of variance was used to compare microbial abundances between groups, followed by post-hoc Tukey’s honest significant difference test for pairwise multiple comparisons. Statistical significance was set at p<0.05. Pearson’s correlation coefficient (R) and p-values obtained from simple linear regression were used to evaluate the correlation between microbial abundance and body weights.

## RESULTS

### Animal growth

**Figure 1:**
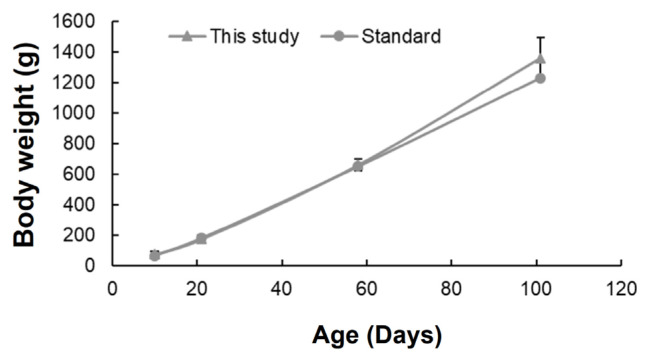
Chicken body weights at different growth stages. Comparison of body weight between standard Hy-line Brown chickens and those in this study. Standard Hy-line Brown weight indicates the average growth provided by the management guide. The average values for each chicken were presented in this study. Error bars indicate standard deviations.

The body weights of chickens rapidly increased as they grew. The trend was similar to that of the standard body weight of Hy-line Brown chickens (Figure 1). The chicken body weights at different growth stages were 74.94±15.71, 174.31±22.65, 660.55±37.86, and 1358.15±133.99 g at 10, 21, 58, and 101 days, respectively.

### Sequencing statistics of the gut microbiome

After sequencing the V4 region of 16S rRNA and performing quality control of the sequences, 3,082,756,756 sequences (mean = 25,906±22,690) were obtained. Each fecal and ileal content sample generated 15,020±6,543 and 48,236±27,359 average reads, respectively. The reads generated in fecal samples of different growth stages were 15,031±7,562 on day 10; 13,178±4,869 on day 21; 13,591±6,329 on day 58; and 18,278 ±6,336 on day 101. Similarly, for the ileal contents at different growth stages, the reads generated were 31,379±11,301 on day 10; 32,479±22,727 on day 21; 61,786±19,238 on day 58; and 65,722±33,101 on day 101.

### Assessing the community of gut microbiota using 16S rRNA sequencing

**Figure 2:**
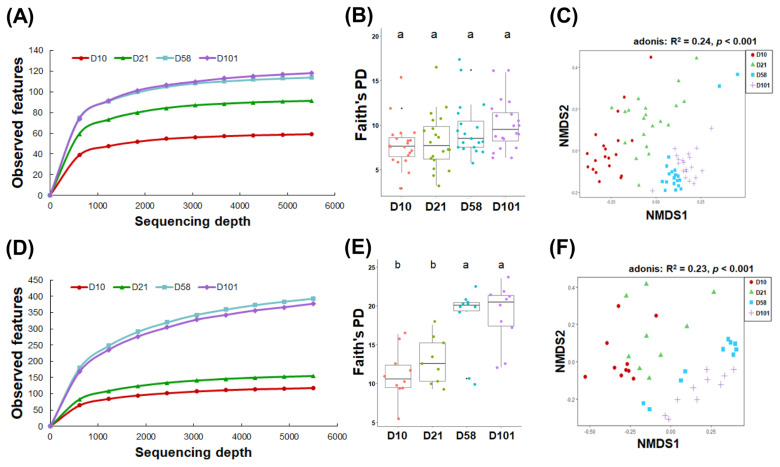
Microbial communities in the feces and ileal contents between the growth stages. Rarefaction curves of observed features in feces (A) and ileal contents (D). Comparison of alpha diversity based on the Faith’s phylogenetic diversity in feces (B) and ileal contents (E). One-way analysis of variance with Tukey’s test was used. NMDS was performed on the gut microbiota using the Bray–Curtis dissimilarity across four age groups in feces (C) and ileal contents (F). Adonis statistical tests were performed with 999 permutations. NMDS, nonmetric multidimensional scaling. ^a,b^ Within a figure, different superscript letters indicate significant differences (p<0.05).

The community diversity was first explored to investigate the microbiomes of developing laying hens. Microbial communities in the feces and ileum exhibited distinct patterns of richness and diversity over time. While the richness of the fecal microbiota significantly increased with age at 10 and 21 days and the late stages (58 and 101 days) (p<0.05; Figure 2A), the ileal samples showed a significant increase from the early (10 and 21 days) to late stages (p<0.05; Figure 2D). Furthermore, as measured by Faith’s phylogenetic diversity, microbial diversity of the ileal samples significantly increased with the progression of growth stages, whereas no significant change was observed in fecal microbial diversity (p<0.001; Figure 2B).

NMDS using the Bray–Curtis dissimilarity method was used to visually represent the changes in the composition of the gut microbiome, which changed with growth. The NMDS plot revealed that the fecal and ileal samples could be categorized into three discrete groups based on their microbial composition: 10 days, 21 days, and late stages (58 and 101 days). Adonis statistical analysis indicated a significant association between the growth stage of chickens and intestinal microbiota composition (feces: R^2^ = 0.24, p<0.001; ileal contents: R^2^ = 0.23, p<0.001; Figure 2C and 2F).

### Relative abundance of gut microbiota at different growth stages

**Figure 3:**
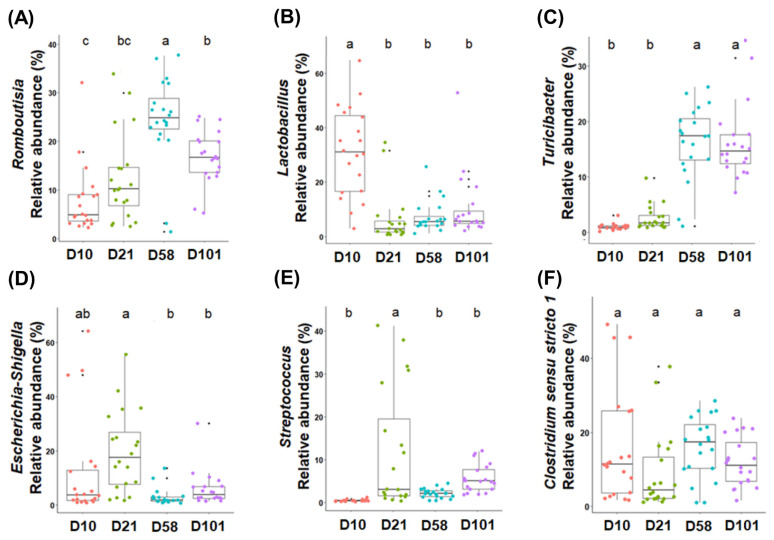
The six most abundant taxonomic genera are colored according to their growth stages in the feces. One-way analysis of variance was used, followed by post-hoc Tukey’s HSD test for pairwise multiple comparisons. HSD, honest significant difference. ^a–c^ Within a figure, different superscript letters indicate significant differences (p<0.05).

**Figure 4:**
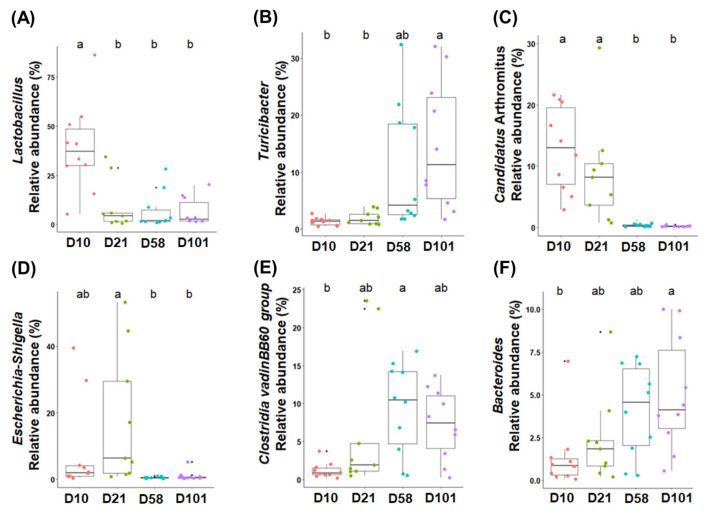
The six most abundant taxonomic genera are colored according to their growth stages in the ileal contents. One-way analysis of variance was used, followed by post-hoc Tukey’s HSD test for pairwise multiple comparisons. ^a,b^ Within a figure, different superscript letters indicate significant differences (p<0.05). HSD, honest significant difference.

Differential analysis was performed to characterize the microbiota in detail to evaluate the relative abundances of all phyla and genera (Tables 2 and 3). The three major phyla in feces and ileal contents were Firmicutes, Proteobacteria, and Bacteroidota. Firmicutes had the highest relative abundance in both groups, and that of Proteobacteria significantly increased with growth and peaked at day 21. Although no significant difference in the abundance of Bacteroidota was observed in fecal samples, it significantly increased in the ileal samples. The major microorganisms identified in the feces at the genus level were *Romboutsia*, *Lactobacillus*, *Streptococcus*, *Clostridium sensu stricto I*, *Escherichia-Shigella*, and *Turicibacter* (Figure 3). The relative abundance of *Romboutsia* gradually increased with growth and peaked at day 58, whereas that of *Lactobacillus* was highest on day 10 and decreased with the growth of the chicken (Figure 3A and 3B). *Streptococcus* was most prevalent at day 21, whereas *Clostridium sensu stricto I* showed no significant differences. The abundance of *Escherichia-Shigella* was highest in the early stages and gradually decreased, whereas that of *Turicibacter* showed the opposite correlation. The abundances of *Lactobacillus*, *Turicibacter*, and *Escherichia-Shigella* in the ileal contents showed similar trends to those in the feces (Figure 4B, 4C, and 4D). The abundance of *Clostridia vadin* BB60 group was highest at day 58, and that of *Candidatus Arthromitus* decreased with growth; however, Bacteroides showed an opposite correlation.

**Figure 5:**
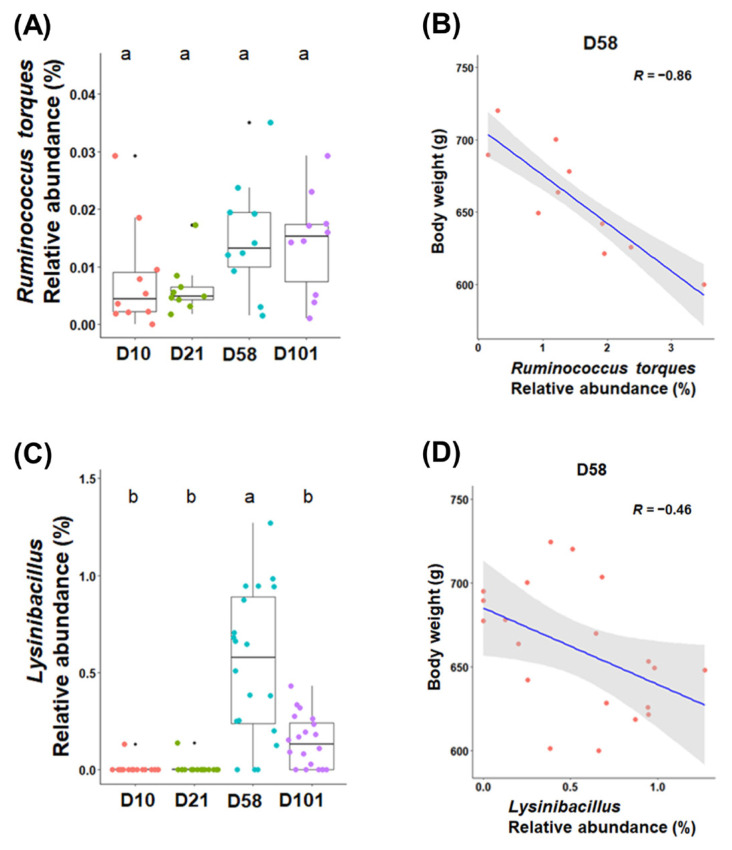
The relative abundances of *Ruminococcus torques* and *Lysinibacillus* are colored according to their growth stages in the feces (C) and ileal contents (A) (p<0.05). The relationship between body weight and relative abundance in the feces (D) and ileal contents (B) at 58 days (p<0.05). Pearson’s correlation coefficient (R) and p-values obtained from simple linear regression were used to evaluate the correlation between the relative abundance and body weight. ^a,b^ Within a figure, different superscript letters indicate significant differences (p<0.05).

Linear regression analysis was conducted to identify the bacterial taxa correlated with body weight in different sampling sections of chickens. For the correlation between genera and body weight, in particular, the abundances of *Ruminococcus torques* (R = −0.86, p<0.001) and *Lysinibacillus* (R = −0.46, p<0.05) were negatively correlated with body weight (Figure 5).

### Metagenomics pathways

**Figure 6:**
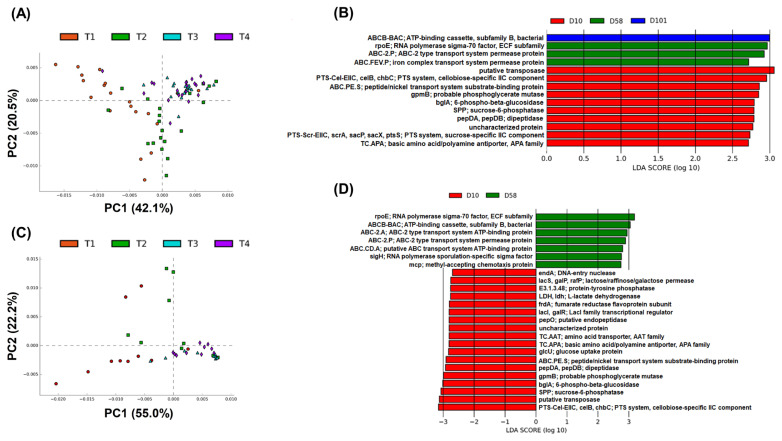
Different functions predicted by PICRUSt at the fourth level of KEGG pathways. PCA plot of PICRUSt in feces (A) and ileal contents (C). KEGG pathway from LEfSe analysis in feces (B) and ileal contents (D). KEGG, Kyoto encyclopedia of genes and genomes; PCA, principal component analysis; LEfSe, linear discriminant analysis effect size.

The KEGG pathways were used to compare the functions of gut microbiota at different growth stages. A total of 6,665 and 6,510 KEGG pathways were identified in the feces and ileal contents, respectively. PCA was conducted at level 4 of the KEGG pathways to observe the sample distribution pattern. For each group of samples, the resulting PCA plot revealed three distinct groups in which the samples were clustered (Figure 6A and B).

Next, linear discriminant analysis effect size (LEfSe; linear discriminant analysis [LDA] score >2.7 and p<0.05) was performed. Fourteen KEGG pathways were identified in the feces (Figure 6B). The pathways related to carbohydrate and energy metabolisms and “putative transposase” had significantly high scores on day 10, while those related to “RNA polymerase sigma-70 factor, ECF subfamily,” “ABC-2 type transport system permease protein” and “iron complex transport system permease protein” had significantly high scores on day 58. The pathways related to “ATP-binding cassette, subfamily B, bacterial” had high scores on day 101. Twenty-five KEGG pathways were identified in the ileal contents (Figure 6D). The pathway related to “PTS-Cel-EIIC, celB, chbC; PTS system, cellobiose-specific IIC component” had a significantly low score on day 10; “putative transposase,” “SPP; sucrose-6-phosphatase,” and “bglA; 6-phospho-beta-glucosidase” had LDA scores <3.0. Several nucleotide, carbohydrate, pyruvate, amino acid, and propanoate metabolic pathways showed significantly low scores on day 10. On day 58, “rpoE; RNA polymerase sigma-70 factor, ECF subfamily” and “ABCB-BAC; ATP-binding cassette, subfamily B, bacterial” had LDA scores >3.0. “ABC-2. A; ABC-2 type transport system ATP-binding protein,” “ABC-2. P; ABC-2 type transport system permease protein,” and “ABC.CD.A; putative ABC transport system ATP-binding protein” were related to signaling and cellular processes; “sigH; RNA polymerase sporulation-specific sigma factor” and “mcp; methyl-accepting chemotaxis protein” were related to genetics and signal transduction, respectively.

## DISCUSSION

A longitudinal study was conducted on the gut microbiota of laying hens across four growth stages in two sampling sites. Longitudinal study refers to a study that tracks or observes the same subject over a long period. Previous studies have analyzed the gut microbiota of laying hens in the early, middle, and late laying stages [15] and broilers at different growth stages [16]. Nutritional responses, immune system interactions, and pathogen infection have been associated with the gut microbiota of laying hens [17]. Laying hens produce eggs after three months, meaning their breeding period is longer than that of broilers. Analyzing the gut microbiota based on the growth stage is crucial for stable breeding. However, there have been limited gut microbiota studies during detailed growth stages before laying in Hy-line hens. This study was conducted because investigating the gut microbiota before laying rather than after laying was considered necessary.

Alpha diversity was investigated to explore the gut micro biota composition in laying hens. Previous studies reported that microbial diversity increased with growth in broilers [18], laying hens [19], and mice [20]. Similarly, the microbial diversity and richness at 101 days were higher than in other growth stages, regardless of sampling sections. The relationship between growth and microbial diversity is affected by various factors, such as age, feed change, and breeding environment [21,22]. Likewise, microbial diversity significantly increased after feed change, implying that growth and feed change positively correlate with microbial diversity. However, further research is required to determine how feed relates to microbial composition.

NMDS analysis was conducted using Bray–Curtis dissim ilarity to validate the changes in the intestinal microbiota during growth. Samples collected on days 10, 21, 58, and 101 were grouped into four distinct clusters representing different age groups, with early-stage clusters comprising samples from days 10 and 21. In contrast, late-stage clusters comprised samples from days 58 and 101. Late-stage clusters differed from early-stage clusters in terms of age and diet composition. Similar alterations in the intestinal microbiota owing to age and diet have previously been observed [7]. For this study, two diets were administered based on the growth stages. Chicks were fed a starter feed on days 10 and 21, and a well-textured feed with modified nutrient content was provided on days 58 and 101 during preparation for laying. The starter feed comprised large particles with high crude protein and fat contents. In contrast, the well-textured mash diet contained crude fiber and ash at elevated levels in the form of pellets (Table 1). Providing appropriate feed according to different growth stages is crucial in the poultry industry because it affects nutrient availability, egg production, and overall productivity.

Changes in the microbiota of feces and ileal contents de pend on age and diet. The abundance of *Lactobacillus* was highest at day 10 in all groups and gradually decreased as the chickens grew. Broilers fed starter feed showed a similar trend [23]. *Lactobacillus* produces lactic acid in the gut, lowering pH and reducing the abundance of pathogens [24]. A high abundance of *Lactobacillus* in chicks positively affects intestinal health, reducing intestinal permeability and improving gut health [25]. Therefore, *Lactobacillus* in the early stages of chicken growth may positively affect health. *Romboutsia* was predominant in the feces and increased with age, reaching its highest relative abundance at day 58. *Romboutsia* is associated with feed utilization and efficiency in broilers owing to its carbohydrate utilization and fermentation of single amino acids [26]. *Escherichia-Shigella* and *Streptococcus* are partially pathogenic; they showed the highest relative abundances at day 21, which decreased as the chickens grew. *Salmonella* is affected by chicken age and immune system [27]. Likewise, both pathogens are thought to be affected by these factors. The abundance of *Clostridium sensu stricto 1* in feces showed no significant differences between growth stages. This genus includes *Clostridium perfringens* and other pathogenic *Clostridium* species [28]. *Candidatus* Arthromitus is found in the terminal ileum of animals and has unique immunomodulatory properties [29]. The abundance of *Candidatus* Arthromitus decreases as chickens grow, and similar results have been observed in poultry [30]. This potentially occurs because immunity was increased during growth. *Turicibacter* has anti-obesity properties, reduces metabolic stress, and inhibits inflammatory reactions in rats [31]. Feeding well-textured feed in the late stages showed an increase in the body weight of chickens compared to that in the early stages. The study examined the relationship between the microbiota and body weight. *R. torques* exhibited a negative correlation with body weights in fecal samples. This microbiome belongs to the *Clostridium coccoides* (XIVa) group in humans and degrades gastrointestinal mucin [32]. While factors such as feed and environment affect the body weight of chickens, our findings highlight the significant influence of microbiota in this regard [33].

The PCA results of metabolic KEGG pathways were clus tered into three groups regardless of sampling sites: day 10, day 21, and late stage (days 58 and 101). LEfSe analysis revealed that the pathways associated with membrane transport, carbohydrate metabolism, and energy metabolism had high scores in the feces. These pathways are essential for bacterial growth to survive in the gut. “PTS-Cel-EIIC, celB, chbC; PTS system, cellobiose-specific IIC component” scored lowest at 10 days in the ileal contents. This is a major active-transport system for carbohydrates, which catalyzes the phosphorylation of incoming sugar substrates concomitant with their translocation across the cell membrane. Phosphorylation is an essential factor for bacterial growth and is regulated by infection in chickens [34]. We hypothesize that this microbial pathway was developed because chicks are vulnerable to infection. However, further studies are required to uncover the exact differences in metabolisms.

## CONCLUSION

The four growth stages were determined to contribute to gut microbiota changes in laying hens. Similar to previous studies, the results showed that various factors, such as growth stage and feed, are crucial. Among them, it is essential to effectively manage pathogens and beneficial bacteria in the early growth stage because pathogen richness in the early growth stage may lead to a decrease in immunity. The results also suggest that there may be interactions between microbiota and feed. Our results can enhance the understanding of microbiology in the poultry industry and will be valuable data for applied research. The changes in the gut microbiota after laying would be an interesting topic for future research.

## Figures and Tables

**Table 1:** Nutrient composition of experimental diets

Feed type	Starter	Well-textured mash
Crude protein (min) (%)	18.0	15.0
Crude fat (min) (%)	3.0	2.0
Crude fiber (max) (%)	5.5	6.5
Crude ash (max) (%)	8.0	9.0
Calcium (min) (%)	0.8	0.7
Phosphorus (max) (%)	0.9	0.9
Methionine+cystine+MHA (min) (%)	0.8	0.5
Metabolizable energy (min) (Mcal/kg)	2.9	2.6

**Table 2:** Relative abundances of phyla and genera in feces at various growth stages

Taxon	Relative abundance (%)				p-value

	10 d	21 d	58 d	101 d	
Phylum					
Actinobacteriota	0.13±0.16^a^	0.64±0.50^a^	1.72±1.39^b^	2.02±1.33^b^	0.00
Bacteroidota	1.52±3.25	2.21±4.08	1.41±2.01	1.55±1.74	0.82
Cyanobacteria	0.36±0.55^a^	1.74±3.19^b^	0.39±0.60^ab^	0.26±0.39^a^	0.02
Firmicutes	84.62±18.19^ab^	74.29±14.48^a^	91.42±5.33^b^	87.89±6.99^b^	0.00
Patescibacteria	0.01±0.01^a^	0.01±0.02^a^	0.67±1.08^b^	0.18±0.18^a^	0.00
Proteobacteria	12.47±18.68^ab^	20.93±14.88^b^	4.13±3.89^a^	7.91±6.78^a^	0.00
Genus					
*Bacillus*	0.00±0.01	0.03±0.06	0.76±1.88	0.61±1.27	0.43
*Bacteroides*	1.37±3.24	2.11±4.10	1.30±2.01	1.28±1.51	0.77
*Brevibacterium*	0.09±0.07^a^	0.13±0.13^a^	0.89±0.82^b^	1.55±1.24^c^	0.00
*Candidatus Arthromitus*	14.83±10.52^c^	6.56±7.15^b^	0.96±0.58^a^	0.53±0.32^a^	0.00
*Cellulosilyticum*	1.31±1.40^b^	0.47±0.60^a^	0.49±0.48^a^	0.39±0.30^a^	0.00
*Clostridium sensu stricto 1*	16.17±15.35	8.93±10.54	15.85±8.42	11.91±6.63	0.11
*Corynebacterium*	1.74±4.13^a^	1.47±1.69^a^	1.27±0.96^a^	4.92±4.11^b^	0.00
*Enterococcus*	2.96±6.08	2.41±1.72	3.39±1.77	4.28±2.60	0.40
*Escherichia-Shigella*	12.01±18.87^ab^	19.59±14.98^b^	2.96±3.21^a^	5.89±6.46^a^	0.00
*Lactobacillus*	31.19±16.40^b^	6.42±9.48^a^	7.43±5.74^a^	10.40±11.68^a^	0.00
*Lactococcus*	0.07±0.08^a^	0.48±0.37^a^	0.99±0.89^b^	1.14±0.74^b^	0.01
*Oscillospira*	1.08±2.78	2.23±3.83	1.90±4.20	1.01±0.83	0.28
*Pseudomonas*	0.13±0.39	0.39±0.65	0.79±1.12	0.69±1.20	0.28
*Romboutsia*	7.91±7.06^a^	12.47±9.09^ab^	24.45±9.13^c^	16.81±5.41^b^	0.00
*Staphylococcus*	0.47±0.35^a^	2.83±3.62^bc^	4.37±3.01^c^	1.14±0.87^ab^	0.00
*Streptococcus*	0.51±0.23^a^	11.93±14.04^b^	2.16±1.10^a^	5.66±3.23^a^	0.00
*Terrisporobacter*	0.53±1.27^a^	2.21±3.67^a^	1.17±1.15^a^	4.74±4.11^b^	0.00
*Turicibacter*	1.00±0.58^a^	2.60±2.25^a^	16.25±6.75^b^	16.24±6.85^b^	0.00

Data is shown as the mean±standard deviations.

One-way analysis of variance with Tukey’s test was used.

a–c Within a row, different superscript letters indicate significant differences (p<0.05).

**Table 3:** Relative abundances of phyla and genera in ileum contents at various growth stages

Taxon	Relative abundance (%)				p-value

	10 d	21 d	58 d	101 d	
Phylum					
Bacteroidota	1.45±2.04^a^	2.53±2.63^a^	4.21±2.53^ab^	6.68±4.05^b^	0.00
Campilobacterota	0.00±0.00^a^	0.00±0.01^a^	0.00±0.00^a^	0.15±0.20^b^	0.00
Firmicutes	85.17±12.38^ab^	74.62±16.94^a^	88.80±4.68^b^	85.03±5.99^ab^	0.04
Patescibacteria	0.06±0.05^a^	0.06±0.03^a^	1.44±1.46^b^	0.56±0.45^ab^	0.00
Proteobacteria	9.16±13.78^ab^	18.83±19.48^b^	2.02±0.89^a^	5.19±2.53^ab^	0.02
Thermoplasmatota	0.00±0.00^a^	0.01±0.01^a^	0.17±0.13^b^	0.10±0.11^ab^	0.00
Genus					
*Alistipes*	0.02±0.02^a^	0.02±0.02^a^	0.02±0.01^a^	1.00±0.66^b^	0.02
*Anaerofustis*	0.00±0.00^a^	0.00±0.00^a^	0.07±0.05^b^	0.00±0.00^a^	0.00
*Bacteroides*	1.38±2.03^a^	2.39±2.64^ab^	4.06±2.67^ab^	5.04±3.35^b^	0.02
*Brachybacterium*	0.03±0.05^a^	0.02±0.04^a^	0.11±0.10^a^	0.11±0.10^a^	0.02
*Brevibacterium*	0.03±0.04^a^	0.02±0.03^a^	0.11±0.09^ab^	0.15±0.13^b^	0.00
*Candidatus* Arthromitus	12.86±6.94^b^	8.98±8.65^b^	0.32±0.20^a^	0.19±0.09^a^	0.00
*Clostridia vadinBB60 group*	1.22±1.05^a^	6.60±9.39^ab^	9.37±6.05^b^	7.32±4.63^ab^	0.03
*Escherichia-Shigella*	8.42±14.03^ab^	17.79±19.97^b^	0.53±0.26^a^	1.07±1.51^a^	0.01
*Lactobacillus*	38.89±22.37^b^	9.33±12.83^a^	6.85±9.33^a^	6.49±7.01^a^	0.00
*Lactococcus*	0.01±0.01^a^	0.00±0.01^a^	0.05±0.05^ab^	0.08±0.06^b^	0.00
*Odoribacter*	0.00±0.00^a^	0.00±0.00^a^	0.00±0.00^a^	0.04±0.03^b^	0.00
*Oscillospira*	0.01±0.05^a^	0.03±0.05^a^	0.22±0.15^b^	0.19±0.14^b^	0.00
*Phascolarctobacterium*	0.07±0.03^a^	0.10±0.05^a^	1.63±1.18^b^	1.59±1.18^b^	0.00
*Ruminococcus*	0.07±0.07^a^	0.14±0.13^a^	1.69±1.11^b^	0.55±0.30^a^	0.00
*Streptococcus*	0.35±0.20^a^	5.90±6.96^b^	0.69±0.43^a^	3.31 ± 3.40^ab^	0.01
*Turicibacter*	1.35±0.69^a^	1.98±1.20^a^	10.81±10.98^ab^	14.68 ± 11.34^b^	0.00

Data is shown as the mean±standard deviations.

One-way analysis of variance with Tukey’s post-hoc test was used.

a,b Within a row, different superscript letters indicate significant differences (p<0.05).

## SUPPLEMENTARY MATERIAL

**Supplementary Table S1**. Sampling data for each growth stage

## References

[1] Mottet A, Tempio G (2017). Global poultry production: current state and future outlook and challenges. World’s Poult Sci J.

[2] Pan D, Yu Z (2014). Intestinal microbiome of poultry and its interaction with host and diet. Gut Microbes.

[3] Dahl WJ, Mendoza DR, Lambert JM (2020). Diet, nutrients and the microbiome. Progress in Molecular Biology and Translational Science.

[4] Nowland TL, Plush KJ, Barton M, Kirkwood RN (2019). Development and function of the intestinal microbiome and potential implications for pig production. Animals.

[5] McLoughlin S, Spillane C, Claffey N (2020). Rumen microbiome composition is altered in sheep divergent in feed efficiency. Front Microbiol.

[6] Adedokun SA, Olojede OC (2019). Optimizing gastrointestinal integrity in poultry: the role of nutrients and feed additives. Front Vet Sci.

[7] Han GG, Lee JY, Jin GD (2018). Tracing of the fecal microbiota of commercial pigs at five growth stages from birth to shipment. Sci Rep.

[8] Khan S, Moore RJ, Stanley D, Chousalkar KK (2020). The gut microbiota of laying hens and its manipulation with prebiotics and probiotics to enhance gut health and food safety. Appl Environ Microbiol.

[9] Jha R, Das R, Oak S, Mishra P (2020). Probiotics (direct-fed microbials) in poultry nutrition and their effects on nutrient utilization, growth and laying performance, and gut health: A systematic review. Animals.

[10] Han GG, Lee JY, Jin GD (2017). Evaluating the association between body weight and the intestinal microbiota of weaned piglets via 16S rRNA sequencing. Appl Microbiol Biotechnol.

[11] Bolyen E, Rideout JR, Dillon MR (2019). Reproducible, interactive, scalable and extensible microbiome data science using QIIME 2. Nat Biotechnol.

[12] Martin M (2011). Cutadapt removes adapter sequences from high-throughput sequencing reads. EMBnet J.

[13] Callahan BJ, McMurdie PJ, Rosen MJ, Han AW, Johnson AJA, Holmes SP (2016). DADA2: High-resolution sample inference from Illumina amplicon data. Nat Methods.

[14] Langille MG, Zaneveld J, Caporaso JG (2013). Predictive functional profiling of microbial communities using 16S rRNA marker gene sequences. Nat Biotechnol.

[15] Van Goor A, Redweik GA, Stromberg ZR, Treadwell CG, Xin H, Mellata M (2020). Microbiome and biological blood marker changes in hens at different laying stages in conventional and cage free housings. Poult Sci.

[16] Glendinning L, McLachlan G, Vervelde L (2017). Age-related differences in the respiratory microbiota of chickens. PLoS One.

[17] Ricke SC (2021). Strategies to improve poultry food safety, a landscape review. Ann Rev Anim Biosci.

[18] Xi Y, Shuling N, Kunyuan T (2019). Characteristics of the intestinal flora of specific pathogen free chickens with age. Microb Pathog.

[19] Liu Y, Yan T, Ren Z, Yang X (2021). Age-associated changes in caecal microbiome and their apparent correlations with growth performances of layer pullets. Anim Nutr.

[20] Yang Y, Chen T, Zhang X, Wang X (2021). Age-related functional changes of intestinal flora in rats. FEMS Microbiol Lett.

[21] Chen S, Xiang H, Zhang H (2019). Rearing system causes changes of behavior, microbiome, and gene expression of chickens. Poult Sci.

[22] Kraimi N, Dawkins M, Gebhardt-Henrich SG (2019). Influence of the microbiota-gut-brain axis on behavior and welfare in farm animals: a review. Physiol Behav.

[23] Singh Y, Ravindran V, Wester T, Molan A, Ravindran G (2014). Influence of feeding coarse corn on performance, nutrient utilization, digestive tract measurements, carcass characteristics, and cecal microflora counts of broilers. Poult Sci.

[24] Wu X, Wen Z, Hua J (2019). Effects of dietary inclusion of Lactobacillus and inulin on growth performance, gut microbiota, nutrient utilization, and immune parameters in broilers. Poult Sci.

[25] Wu Z, Yang K, Zhang A (2021). Effects of Lactobacillus acidophilus on the growth performance, immune response, and intestinal barrier function of broiler chickens challenged with Escherichia coli O157. Poult Sci.

[26] Madigan-Stretton J, Mikkelsen D, Soumeh EA (2021). Multienzyme super-dosing in broiler chicken diets: The implications for gut morphology, microbial profile, nutrient digestibility, and bone mineralization. Animals.

[27] Song J, Li Q, Everaert N (2020). Effects of inulin supplementation on intestinal barrier function and immunity in specific pathogen-free chickens with Salmonella infection. J Anim Sci.

[28] Lakshminarayanan B, Harris HM, Coakley M (2013). Prevalence and characterization of Clostridium perfringens from the faecal microbiota of elderly Irish subjects. J Med Microbiol.

[29] Jonsson H, Hugerth LW, Sundh J, Lundin E, Andersson AF (2020). Genome sequence of segmented filamentous bacteria present in the human intestine. Commun Biol.

[30] Richards-Rios P, Fothergill J, Bernardeau M, Wigley P (2020). Development of the ileal microbiota in three broiler breeds. Front Vet Sci.

[31] Zhou W, Xu H, Zhan L, Lu X, Zhang L (2019). Dynamic development of fecal microbiome during the progression of diabetes mellitus in Zucker diabetic fatty rats. Front Microbiol.

[32] Gyawali I, Zeng Y, Zhou J (2022). Effect of novel Lactobacillus paracaesi microcapsule on growth performance, gut health and microbiome community of broiler chickens. Poult Sci.

[33] Rajilić-Stojanović M, De Vos WM (2014). The first 1000 cultured species of the human gastrointestinal microbiota. FEMS Microbiol Rev.

[34] Kogut MH, Genovese KJ, Byrd JA (2022). Chicken-specific kinome analysis of early host immune signaling pathways in the cecum of newly hatched chickens infected with salmonella enterica serovar enteritidis. Front Cell Infect Microbiol.

