# Supplementary material for: A comprehensive longitudinal study of gut microbiota dynamic changes in laying hens at four growth stages prior to egg production
Source: Anim Biosci. 2023 Oct 21;36(11):1727–37. doi: 10.5713/ab.23.0271 (PMC10623045; doi:10.5713/ab.23.0271)
Supplement: Supplementary file 1 [file ab-23-0271-Supplementary-Table-1.pdf]

Suppplemental Table S1. Sampling data for each growth stage.

Age	Sampling dates[1]	Number of feces	Number of ileal contents	Feed
10	09/2021	20	10	Starter
21	09/2021	20	9	Starter
58	10/2021	20	10	Well-textured mash
101	12/2021	20	10	Well-textured mash

[1] Dates the laying hens were sampled in month/year.
